# Nanoindentation Study of Calcium-Silicate-Hydrate Gel via Molecular Dynamics Simulations

**DOI:** 10.3390/nano13182578

**Published:** 2023-09-18

**Authors:** Hang Yin, Xuefeng Wang, Haifeng Qin, Shijie Wang, Kun Cai

**Affiliations:** 1 College of Water Conservancy and Civil Engineering, Shandong Agricultural University, Tai’an 271018, China; yinh@sdau.edu.cn (H.Y.);; 2 School of Science, Harbin Institute of Technology, Shenzhen 518055, China

**Keywords:** calcium-silicate-hydrate, nanoindentation, mechanical property, uniaxial test, molecular dynamics

## Abstract

The mechanical properties of calcium-silicate-hydrate (C-S-H) gels in cementitious materials are mainly realized by nanoindentation experiments. There is limited research on the dynamic response of the molecular structure of C-S-H under nanoindentation conditions. This study simulated the nanoindentation on the C-S-H gel samples by the molecular dynamics method considering the essential factors of modeling and loading process. The results demonstrate that the averaged elastic moduli we obtained had slight differences from those by experiments. In contrast to the experimental results, the gels showed bi-modulus and transverse isotropic with the material principal direction perpendicular to the C-S-H layers. The modulus in a direction increased with the loading speed, which indicates that C-S-H behaves viscous due to the water motion in the sample and the propagation of stress wave. The saturation of water influenced the moduli differently because more water in C-S-H will reduce the polymerization of silicon chains and then weaken the local stiffness. The conclusions provide a deeper understanding of the mechanism on the unique mechanical response of C-S-H gels.

## 1. Introduction

Calcium silicate hydrate (C-S-H) is the major hydration product of cementitious material. The mechanical properties of the cement depend on the distribution of C-S-H. According to different microstructural characterization methods and cement hydration processes, C-S-H typically exhibits diverse multiscale morphology [1,2]. Hence, the structural and mechanical analysis of C-S-H at micro/nano scale attracted much attention in recent years [3]. The investigations can be used to develop the concrete with ultra-high performance and reveal the instinct of drying shrinkage and creep mechanism [4] for instance. Nanoindentation technique has been widely applied in the mechanical tests of cementitious materials for over a decade, and it has developed sophisticated methods in terms of sample preparation and analysis [5].

In the experimental studies, Constantinides and Ulm [6] first validated the existence of the high-density (HD) and low-density (LD) C-S-H in cement using the nanoindentation technique. They also investigated the influences of the two types of gel on the elasticity of the cement, and proposed a two-step homogenization approach to predict the elasticity of the cement at the macroscale via nanoindentation. Subsequently, they [7] applied a novel grid-indentation technique to test the mechanical properties of the hardened cement paste microstructure C-S-H, and processed a large amount of measured point data using deconvolution techniques. It was demonstrated that both stacking densities C-S-H have the mechanical properties of nanoparticles and their mechanical behavior is determined by the contact forces between the particles, independent of the mineral composition properties. This behavior may be caused by the random precipitation of C-S-H particles during the hydration reaction and the percolation of more than 50% of the stacking density. In subsequent studies, researchers have combined nanoindentation with other microstructure or phase characterization techniques such as scanning electron microscope (SEM) [8,9,10,11,12,13] and atomic force microscope (AFM) [14], thus providing a method to relate mechanical properties to microstructure. These applications of nanoindentation provided some vital information and insight into the mechanical properties of the microstructure of cementitious materials [15].

As a matter of fact, experimental approaches have difficulty in fully capturing the deformation process during indentation, which is paramount for understanding the correlation between the microstructure and mechanical properties of C-S-H. Leveraging the instantaneous response with atomistic details [16] offered by Molecular Dynamics (MD) simulations, they are widely employed in the study of C-S-H’s mechanical properties based on the molecular model proposed by Pellenq et al. [17]. It is typically derived by modifying the layered structure of Tobermorite or Jennite. MD simulations primarily focus on investigating the mechanical [3,18] and thermal properties [19,20] of the extended periodic structure of C-S-H layered models. Additionally, these simulations may involve expanding the interlayer distances to explore the characteristics of gel pores at the nanoscale [21,22]. The reliability of such layered structures has been confirmed through first-principles investigations, with calculated Young’s modulus showing close agreement with nanoindentation test results [23]. Mutisya et al. [24] further validated that the empirical force field established based on clay models yields thermodynamic quantities consistent with first-principles computations. These findings form a solid foundation for utilizing MD in the study of C-S-H mechanical properties.

In general, the investigation of mechanical properties is typically achieved through uniaxial tension/compression [25,26], shear [27,28], or nanoindentation simulations [29,30]. Hou et al. [31,32,33,34,35] conducted a series of uniaxial tension studies. The results have shown that fracture in the x and y directions exhibits good ductility, but the stress suddenly decreases after yielding in the z direction, indicating brittle damage of the model. The connection between layers is the weakest due to the instability of the hydrogen bond network and the shielding of calcium ions in the z-direction, leading to lower tensile strength and Young’s modulus. Fan et al. [36] further investigated the interfacial mechanical properties between C-S-H gel particles. It was observed that the orientation of the layered structure remains a primary factor influencing the mechanical performance, while the moisture content at the interface had a relatively minor impact on the results. Manzano et al. [27] discovered, through shear simulations, that interlayer water serves as the preferential site for shear strain generation. This finding paves the way for investigating the creep mechanisms in cementitious materials. Subsequent studies [37,38] have demonstrated that by employing periodic shear and applying load–unload cycles, MD simulations can yield results consistent with creep theory models and macroscopic experiments. Researchers have also introduced Ca/Si ratios [32,39], voids [34], water molecules [40], and defects [41,42] for a more detailed study of C-S-H.

In general, the application of MD simulations in studying the mechanical properties of C-S-H allows for the analysis of the underlying mechanisms through aspects such as atomic structural distribution, chemical bonding, and the strength of hydrogen bonds. This approach provides rich details on the dynamic evolution of mechanical behavior, establishing a closer connection between experimental observations and theoretical studies. However, nanoindentation, as one of the main experimental methods for studying the mechanical properties of C-S-H gel, has been less directly applied to analyze the impact of mechanical properties in the C-S-H molecular model. In this study, we aimed to apply nanoindentation process with MD simulation to further elucidate mechanical properties and deformation behaviors of atomic C-S-H model. The essential factors including loading speed, the size of indenter, the maximum indentation depth, and saturation of water in model will be considered when testing the model along three normal directions of the material.

## 2. Simulation Methods

### 2.1. Modeling and Simulation Details

#### 2.1.1. C-S-H Cell Model

**Figure 1:**
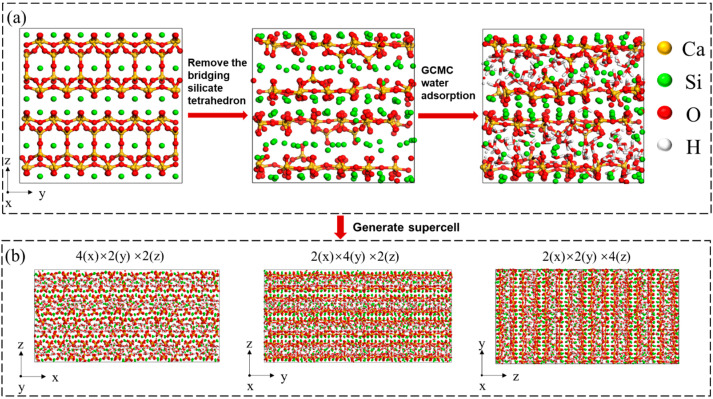
C-S-H model construction process. (**a**) Orthogonal modified cell based on initial Tobermorite 1.1 nm structure. (**b**) Uniaxial tensile/compression simulation models of C-S-H supercells.

The model shown in Figure 1 was built based on the method proposed by Pellenq et al. [17]. Firstly, the monoclinic cell of Tobermorite with interlayer spacing of approximately 1.1 nm was expanded to an orthogonal modified cell with the size of 2.24 nm (x) × 2.22 nm (y) × 2.26 nm (z) (Figure 1a). A part of SiO_2_ (neutral) molecules were then randomly removed for satisfying the distributions of *Q* species obtained from nuclear magnetic resonance (NMR) testing [43]. For example, the distributions of *Q*_0_, *Q*_1_, and *Q*_2_ reached 12.94%, 65.88%, and 21.18%, respectively. Since the effect of oxhydryl (-OH group) on the mechanical property of C-S-H is small [17,44], it was not considered in the present work. Secondly, Grand Canonical Monte Carlo (GCMC) simulation of the water adsorption was operated at 300 K. The chemical formula of the saturated C-S-H structure is (CaO)_1.69_(SiO_2_)·1.79H_2_O, which was very close to the (CaO)_1.7_(SiO_2_)_·_1.8H_2_O obtained by the small angle neutron scattering (SANS) test [45]. Finally, a further 2000 ps relaxation at the NPT (N: number of atoms; P: pressure; T: temperature of system) ensemble (isothermal isobaric ensemble) was conducted to achieve the structures of C-S-H gel in equilibrium states.

#### 2.1.2. Uniaxial Tensile/Compression Modeling and Simulation Details

Before indentation simulation, we evaluated the uniaxial properties of the C-S-H model (Figure 1b). For example, when testing the properties along the z-direction, we first created a supercell containing 2(x) × 2(y) × 4(z) modified cells for uniaxial tests. The super-cell was then relaxed under the isothermal isobaric (NPT) ensemble with T = 300 K and P = 0 [29,33,35], and the equilibrium configuration was achieved after 10 ps of relaxation. Finally, the C-S-H structure was elongated in the z-direction with strain rate of 0.008/ps under the NPT ensemble with Px = Py = 0. Pz indicates the internal stress σ_zz_ along the z-direction. The time integration of the motion equation was performed by using the Verlet leapfrog algorithm with a time step of 0.1 fs. The electrostatic Coulombic interactions were evaluated by the Ewald summation method with a cutoff of 1.0 nm.

The mechanical properties of C-S-H gel exhibit anisotropic due to their molecular structure at nanoscale, while the cement behaves isotropic at macroscale [7,46]. To quantify the anisotropy at the nanoscale, we used the Reuss–Voigt–Hill (R-V-H) average method [47] to predict the indentation modulus *M*_R-V-H_ [44] and Poisson’s ratio μ of C-S-H, i.e.,

(1) $$M_{R\text{-}V\text{-}H}=4G\frac{3K+G}{3K+4G},$$

(2) $$\mu_{R\text{-}V\text{-}H}=\frac{1}{2}\left(1-\frac{3G}{3K+G}\right),$$

where *K* and *G* are the mean values of bulk and shear moduli calculated by Voigt method [48] and Reuss method [49], respectively (Hill method).

#### 2.1.3. Nanoindentation Model

In the supercell for nanoindentation of C-S-H, there are the same number of unit cells in the three dimensions (x-y-z). To avoid the boundary effects, along the indentation direction the supercell in simulation was divided into three segments. Bottom-up, they were the bottom layer (B-L) with the thickness of 1/9 supercell height, the thermostat layer (T-L) with the thickness of 2/9 supercell height, and the Newtonian layer (N-L) with the thickness of 2/3 supercell height. The atoms in the B-L were confined to have horizontal motion. The atoms in the T-L were used to control the temperature at 300 K. The energy transition between the T-L and N-L occurs when the atoms in the N-L is compressed by the indenter. Throughout the entire loading and unloading process, the system maintained energy balance. The variation in total energy (kinetic energy + potential energy) stemmed from the work carried out by the indenter on the system and the total heat extracted from the system by the thermostat at constant temperature [50,51].

In each simulation, the super-cell was first relaxed to reach an equilibration state before nanoindentation. In relaxation, the system energy was minimized using the conjugate gradient algorithm. The system was relaxed for 10 ps under the isothermal isobaric (NPT) ensemble with T = 300 K, P = 0, and periodic boundary conditions along the three dimensions. Second, the indentation direction was changed to the free boundary after the 10 ps relaxation. The system was further equilibrated under the canonical ensemble (NVT) with T = 300 K for another 10 ps. Third, the atoms in the B-L were fixed and the remained part of the system was relaxed under NVT ensemble with T = 300 K for 10 ps. Finally, the indenter moved toward the supercell with a constant velocity (*v*) for starting indentation (loading process) and returned to its initial position (unloading process) when its displacement reach the specified maximum indentation depth (*h*_max_). The loading speed of indenter was set with *v* = 50 m/s unless otherwise noted. It is a common choice for MD simulation during the indentation process for concept study [29].

In the present study, a virtual spherical indenter [52,53,54] was adopted for indentation. In the supercell, the atoms near the indenter will be loaded with the force yields:

(3) $$f_{i}=\left\{\begin{array}{l} -k{\left(R-r_{i}\right)}^{2}\\ 0 \end{array}\right.\begin{array}{c} \;\mathrm{when}\;R>r_{i},\\ \mathrm{others},\; \end{array},$$

where *k* is a constant describing the rigidity of the indenter, herein, equals 3 eV/Å^3^ [55]. *R* is the radius of the indenter, and *r_i_* is the distance between atom *i* and the centroid of the spherical indenter. If no further instructions are given, the radius of indenter remains 2 nm in the simulations.

**Figure 2:**
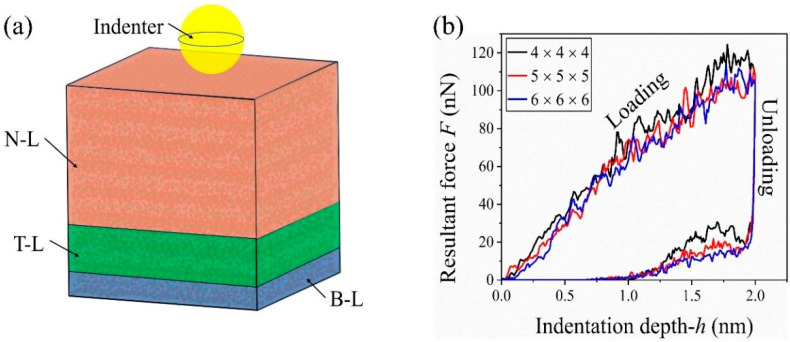
(**a**) Schematic model for nanoindentation simulation of C-S-H sample using a virtual spherical indenter. In a MD simulation, the supercell contains three layers, i.e., bottom layer (B-L) for applying displacement constraint, thermostat layer (T-L) for controlling the temperature, and Newtonian layer (N-L) for showing the indentation-induced deformation. (**b**) Orthorhombic cell containing two C-S-H nanolayers.

Both the Hertz method [56] and the Oliver–Pharr method [57,58] are available to calculate the indentation modulus of a material under indentation. In this study, the Hertz theory was used to calculate the indentation modulus, i.e.,

(4) $$M=\frac{3F}{4\sqrt{Rh^{3}}},$$

where *F* is the resultant force provided by the indenter (Figure 2a), *h* is the indentation depth in the loading stage.

Simultaneously, the hardness of the material can be calculated by the following equation:

(5) $$H=\frac{F_{\max}}{A}=\frac{F_{\max}}{\pi \left(2Rh_{\max}^{}-h_{\max}^{2}\right)},$$

where *A* is the projected contact area between the indenter and the specimen, *h*_max_ is the maximum indentation depth at the end of loading, accordingly, *F*_max_ is the maximum of *F*.

The relationship between the indentation modulus and the Young’s modulus of the specimen reads

(6) $$\frac{1}{M}=\frac{1-\mu_{S}^{2}}{E_{S}}-\frac{1-\mu_{I}^{2}}{E_{I}},$$

where *E*_S_ and *E*_I_ are the Young’s moduli of the specimen and the indenter, respectively, and *μ*_S_ and *μ*_I_ are their Poisson’s ratios. As the indenter is considered as a rigid, i.e., *E*_I_ is infinite, and therefore, the Young’s modulus of the specimen reads

(7) $$E_{S}=M\left(1-\mu_{S}^{2}\right),$$

Accordingly, the Poisson’s ratio of the specimen is obtained by uniaxial test.

To reveal the size effect, herein, three different supercells were built and tested. A supercell may contain 4, 5, or 6 unit cells in each dimension, i.e., the supercell reads 4 × 4 × 4 (with the size of 8.8 nm × 8.9 nm × 9.0 nm), 5 × 5 × 5 (11.0 nm × 11.2 nm × 11.2 nm), or 6 × 6 × 6 (13.2 nm × 13.4 nm × 13.5 nm) model. The loading-displacement curves of the three supercells shown in Figure 2b demonstrate that the curves have a slight difference. Hence, the size effect can be avoided when each side of the supercell is longer than ~9 nm. Considering the expensive cost on computation, the 4 × 4 × 4 supercell will be used in the following discussions.

### 2.2. Interatomic Potentials

The MD simulations in this study were performed on the open-source code ‘Large-scale Atomic/Molecular Massively parallel Simulator’ (LAMMPS) developed by Plimpton [59], In the above simulation (Figure 2b), the interatomic interaction was evaluated by the ClayFF force field [60], which has been widely used in C-S-H-related simulations [24,35,61,62,63,64,65]. The force field can describe the metal–oxygen ionic interactions associated with hydrated phases. All atoms were charged points having complete translational freedoms. The hybrid interactions were illustrated by a 12-6 type Lennard-Jones (LJ) potential together with electrostatics. The parameters in the potential function were obtained by optimizing the mineral structures and the partial atomic charges determined by the periodic density functional-theory-based calculation of simple oxide, hydroxide, and oxyhydroxide compounds with well-defined structures. In this study, a flexible SPC (simple point charge)-based water model was adopted to evaluate the responses of the water and hydroxyl. The short-range (van der Waals) interaction reads

(8) $$\Pi_{ij}=4\varepsilon_{ij}\left[{\left(\frac{\sigma_{ij}}{r_{ij}}\right)}^{12}-{\left(\frac{\sigma_{ij}}{r_{ij}}\right)}^{6}\right],$$

where *ε_ij_* and *σ_ij_* are listed in Table 1 for the detailed species in the present model.

## 3. Results and Discussion

### 3.1. Elasticity Obtained by Indentation and Uniaxial Simulation

We first compared the values of Poisson’s ratio of the C-S-H gel tested by different methods. Table 2 lists the details that demonstrate the values had slight differences. For example, it equaled 0.26 by the present uniaxial simulation, which is slightly lower than that by the approximated Reuss–Voigt–Hill method, and slightly higher than that by Le Bellégo [66]. For the C-S-H gel sample, Poisson’s ratio had great dependency on the compressive direction; however, slightly on the tensile direction [32]. It demonstrates that the sample showed anisotropic due to the microstructure of the gel having direction dependency.

**Figure 3:**
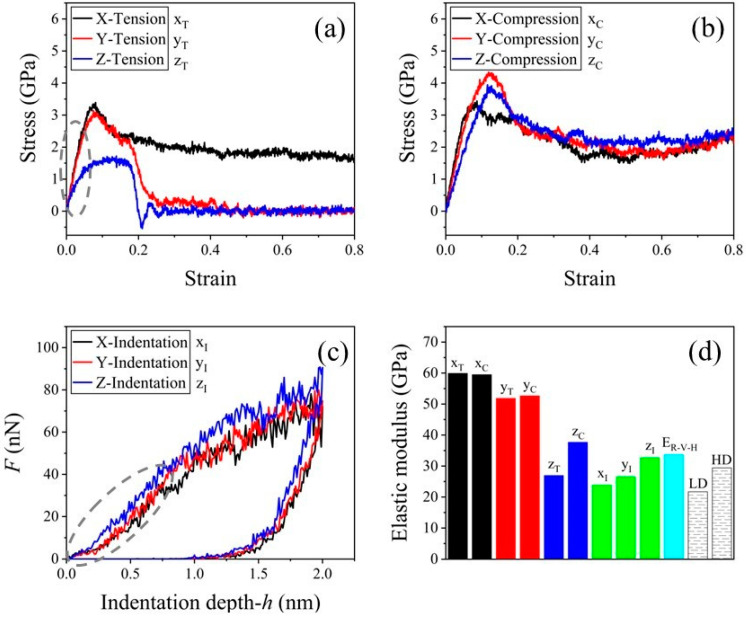
Modulus comparison. (**a**,**b**) The stress–strain curves of the C-S-H supercell stretched or compressed with the speed of 0.05 nm/ps along the three different directions. (**c**) The load-indentation depth curves along the three orthogonal directions. (**d**) Moduli of the C-S-H sample obtained by different methods. Black: X-Tension/Compression, Red: Y-Tension/Compression, Blue: Z-Tension/Compression, Green: X, Y, Z-Indentation, and Cyan: E_R-V-H_. The LD (low density) and HD (high density) data were obtained from the nanoindentation test in experiment [6].

Elastic moduli estimated by both the uniaxial and indentation simulations were also compared. Figure 3a,b show the constitutive curves of the C-S-H sample under either uniaxial tension or compression. Young’s modulus should be evaluated at the initial linear stage of a stress–strain curve, e.g., the segment in the dash-line circle in Figure 3a, while the modulus was predicted using Equation (4) with the indentation force and depth picked at the initial loading stage (circle by dash-line in Figure 3c). In Figure 3d, Young’s modulus of C-S-H gel estimated by the Reuss–Voigt–Hill model is listed, as well. Young’s modulus of LD C-S-H and HD C-S-H samples obtained by tests [6] are given for comparison.

The modulus of the C-S-H by indentation in the z-direction (z_I_) was slightly higher than that of HD but slightly lower than *E*_R-V-H_, whilst *E*x_I_ and *E*y_I_ were between those of the LD and HD samples. Hence, the moduli obtained by nanoindentation were very close to the experimental results. However, the indentation moduli were much lower than those by the uniaxial tests along the x- and y-directions. In peculiar, the C-S-H gel showed heavy bi-modulus behavior [67]. The differences among the moduli in different directions were well aligned with the previous uniaxial MD simulation results [32,39,68]. The bi-modulus behavior when tested in the z-direction was mainly caused by the layout of the C-S-H layers. When the gel was under compression in the z-direction, the water layer supported the compressive stress from its neighboring C-S-H layers. However, the water layer deformed greatly when under tension. Hence, the tensile stiffness was lower than the compressive stiffness in the z-direction.

**Figure 4:**
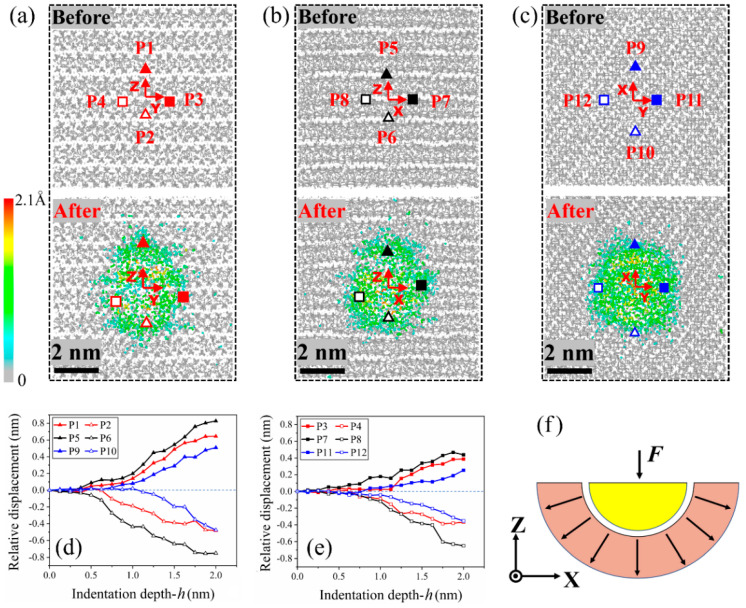
Local deformation at the indentation area. (**a**) Before and after indentation in the x-direction, and (**b**,**c**) indentation in the y- and z-direction. (**d**,**e**) The relative displacement of the four observation points in each case. Negative value means moving opposite to the positive axial direction (the red arrows labeled X, Y, or Z). (**f**) Schematic of the motion of the contact area in an indentation process.

For demonstrating the motion or deformation of the contact area below the indenter, herein, four observing points were marked and their coordinates are listed in Table 3. After releasing the load, the residual displacement fields are shown in Figure 4a–c. The relaxed contacted areas in Figure 4a,b become ellipses indicating that the material behaves orthotropic. The reason is that the silicon chains have larger in-layer deformation than the transverse deformation. The residual displacement filed in Figure 4c was much close to a circle, i.e., the in-plane deformation was uniform, which implies that the material showed isotropic. Hence, the C-S-H gel in this study showed transverse isotropic with the material principle direction perpendicular to the C-S-H layer. The anisotropy can be evaluated by the ratio of *E*z_I_ to *E*x_I_ or *E*y_I_, i.e., *E*z_I_/*E*x_I_ = 32.73/23.88 = 1.37, or *E*z_I_/*E*y_I_ = 32.73/26.58 = 1.23. The Si-O bonds were stronger than the Ca-O bonds, and then introduced the difference between *E*x_I_ and *E*y_I_.

### 3.2. Effect of the Maximum Indentation Depth

**Figure 5:**
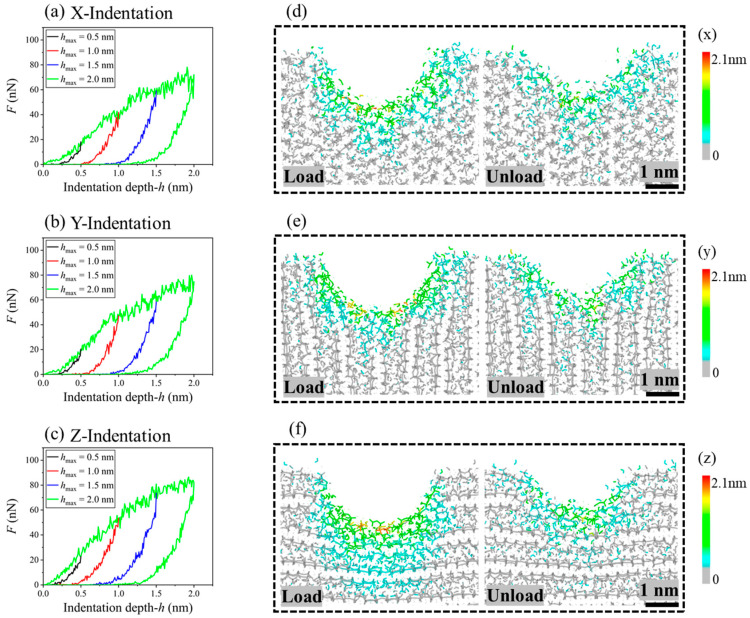
Indentation curves of the C-S-H gel compressed by the indenter with v = 50 m/s at T = 300 K. (**a**–**c**) Indentation in the x-, y-, and z-direction, respectively. (**d**–**f**) Before and after releasing the indenter with the maximum indentation depth of 2 nm in the x-, y-, or z-direction.

When loading depth of the indenter varied from 0.5 nm to 2.0 nm, the maximum of the resultant force in the x-direction increased from 15.51 nN to 68.33 nN, or from 19.52 nN to 74.78 nN in the y-direction, or from 24.51 nN to 82.34 nN in the z-direction. Accordingly, the hardness calculated by Equation (5) changed from 2.82 to 5.44 GPa in the x-direction, from 3.55 to 5.95 GPa in the y-direction, or from 4.46 to 6.56 GPa in z-direction. Hence, it was not feasible to calculate the hardness of the C-S-H gel using an indenter with radius lower than 2 nm. From the curves in Figure 5a–c, we found that the slope of the loading segment decreased gradually when indentation was applied in any direction. This was due to the projected area of indentation being approximately proportional to the square of the indentation depth (Equation (5)).

**Figure 6:**
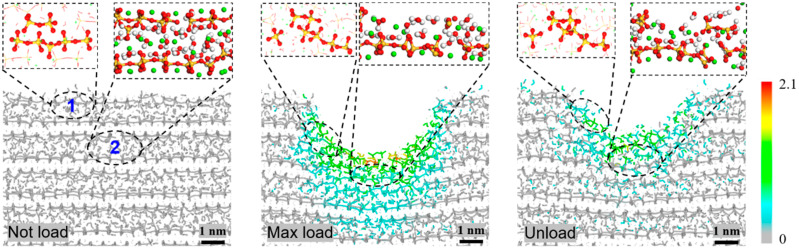
Changes of local bond topologies at the two points (labeled 1 and 2) below the indenter before (Not load) and after loading (Max load) with h_max_ = 2.0 nm at 300 K and after unloading (Unload).

As the indentation depth approached 2.0 nm (load), more atoms right below the indenter had large displacements when indentation was carried out in the z-direction (Figure 5f) than in either the x- or y-direction (Figure 5d,e). After full relaxation (unload), the depth of the residual deformation at the indentation area, in any direction, was higher than 1 nm but lower than the maximum indentation depth. From the local bond topology (Figure 6), one can find that the neighboring C-S-H layers were bonded together at the bottom area when compressed in the z-direction. The new bonds between neighboring layers were mainly on the upper part of the indentation hole.

When observing the local bonds on the two C-S-H layers right below the indenter (Figure 6), we found that bond breakage and generation occurred simultaneously at point 1. Even after unloading, the new bond topology at point 1 did not change. The silicon chains at point 2 broke heavily because they were right below the tip of the indenter (Video S1). Meanwhile, some of the water molecules at point 2 were excluded after loading. Even removing the indenter (unload), the water molecules did not come back due to the complicated layout of the silicate, calcite, and the remaining water molecules. Hence, the residual deformation was obvious. The formation of an amorphous layer in the contact area between the indenter and the substrate is a common phenomenon analogous to contact [51,69,70].

### 3.3. Effect of the Water Saturation in C-S-H

**Figure 7:**
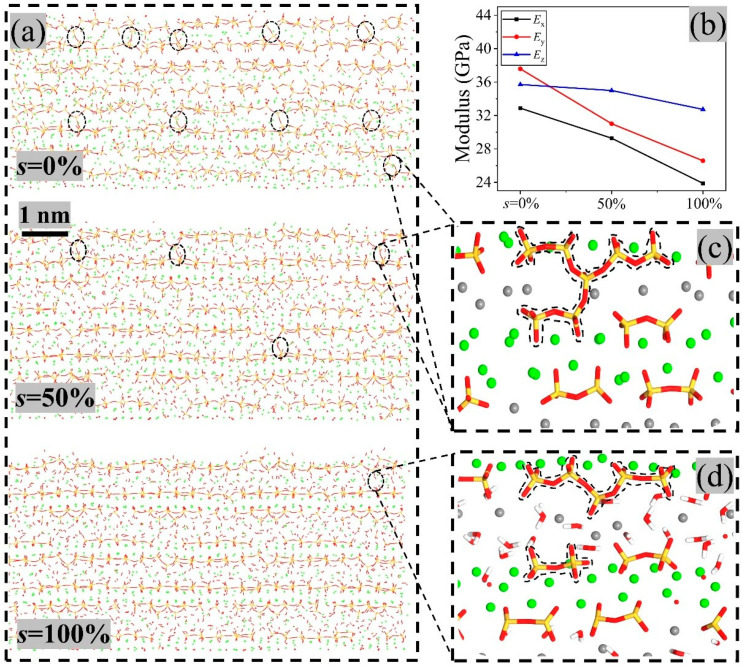
Polymerization of the C-S-H sample (**a**) with different water saturation (s). (**b**) Elastic moduli in different direction of the sample. (**c**) Q3 structure after polymerization between a pentamer (5-polymer) and a dimer (2-polymer). (**d**) Polymerization prevented by water layer.

The water saturation (s) in a cement-based material has a significant effect on its mechanical properties [63]. We calculated the moduli of the sample with three different saturations. Note that the water saturation (s) was calculated by the molar ratio of the number of water in gel to the saturated water number. The results in Figure 7b indicate that the modulus in a direction of the sample decreased with the increasing saturation. For instance, E_x_ decreased from 32.88 GPa at s = 0% to 23.88 GPa at s = 100%. In y-direction, the modulus decreased from 37.58 GPa at s = 0% to 26.58 GPa at s = 100%. The relative differences of the moduli in the x- and y-direction reached ~40%. However, in the z-direction, the elastic modulus decreased slightly from 35.71 GPa at s = 0% to 32.73 GPa at s = 100%. The relative difference was about 9.1%. The reason for this was that the water moved easier when the indenter compressed in the x- or y-direction than compressed in the z-direction. When compressed in the x- or y-direction, the indenter contacted the C-S-H layers and water layers directly. The water molecules between two C-S-H layers moved out of the contact area when the pressure was high enough. However, when compressed in the z-direction, the indenter only contacted the C-S-H layer, which will be supported by the water layer below it. Hence, E_z_ decreased slightly.

The decreasing of moduli was essential to the polymerization degree in the sample. For example, when no extra water molecules were absorbed in the nanostructure, polymerization occurred frequently, which can be verified by comparing the three configurations in Figure 7a. The sample had more *Q*_3_ structures (circled by dash-lines) at *s* = 0% than at *s* = 50%, which can enhance the interlayer bonding strength at z-direction. Note that each *Q*_3_ structure was formed by a pentamer and a dimer (Figure 7c). It hardly appeared in the sample with *s* = 100%. The chance of polymerization between silicon was reduced by the water layers between them. At the solid–fluid interface, the water saturation is positively correlated with the interaction energy [50,71]. When the moisture content reached saturation, the interaction energy peaked, and the phenomenon of cross-layer bonding diminished. The variation of *E*_z_ may suggest that within the internal structure of C-S-H’s layered configuration, the interaction at the solid–fluid interface was slightly weaker than the interlayer bonding strength.

### 3.4. Effect of the Loading Speed

**Figure 8:**
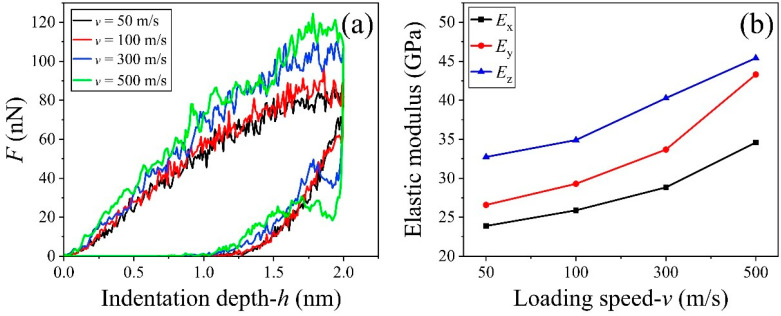
Force-indentation depth curves of the C-S-H sample under a loading speed (v) between 50 and 500 m/s at T = 300 K. (**a**) Loading and unloading curves when compressed in z-direction, (**b**) the elastic moduli of the sample in three different directions at different loading speeds.

In general, the loading speed in a nano-indentation is between 30 and 60 nm/s [72,73]. Considering the effect of computational cost, the loading speed in a MD simulation is set to be 50–400 m/s [55,74,75,76,77]. In this study, we investigated the effect of loading speed considering four cases, i.e., 50, 100, 300, and 500 m/s. As shown in Figure 8a, the resultant force on the indenter was greater at a higher loading speed when it had the same indentation depth. In particular, the peak value of force *F* at 300 m/s in the z-direction was much higher than that at 100 m/s. It indicates that the material behaved obvious viscosity, which was caused by two factors, i.e., the motion of the water molecules in the C-S-H layers (Video S2) and the propagation of stress wave. Briefly, when the loading speed was higher, the water molecules were not expelled from the region under the contact area as soon as the region was compressed; hence, the water molecules contributed more to the local stiffness. Hence, we found that the elastic modulus in any direction increased with the loading speed (Figure 8b), which is consistent with previous studies [78]. For instance, Young’s modulus in the x-direction varied from 23.88 GPa at *v* = 50 m/s to 34.59 GPa at 500 m/s; when tested in the y-direction, it increased from 26.58 GPa at 50 m/s to 43.30 GPa at 500 m/s, or from 32.73 GPa to 45.41 GPa, accordingly.

### 3.5. Effect of the Indenter Radius

**Figure 9:**
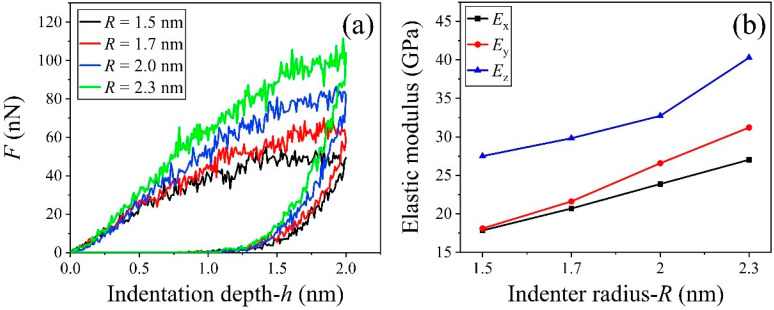
Force-indentation depth curves of the C-S-H sample compressed by the indenters with different radii between 1.5 and 2.3 nm at v = 50 m/s and T = 300 K. (**a**) Loading and unloading curves when compressed in z-direction, (**b**) the elastic moduli of the sample in three different directions and compressed by the indenters with different radii.

In this study, the maximum indentation depth was set to 2.0 nm for all cases. When the radius of the indenter was 1.5 nm, the peak force fluctuated nearby 50 nN when the indentation depth exceeded 1.4 nm (Figure 9a). The reason for this was that the smaller indenter was easier to break the covalent bonds right below the indenter tip and the broken C-S-H layer had a weaker response to the motion of the indenter. Hence, the peak force did not increase with the indentation depth when the depth was higher than 1.4 nm (slightly lower than *R* = 1.5 nm). As a bigger indenter was used, the resultant force increased with the indentation depth before it reached the peak value, which increased with the radius of indenter.

We collected the elastic moduli (Figure 9b) and found the modulus in z-direction (*E*_z_) was much higher than the in-plane moduli (*E*_x_ and *E*_y_) when using the same indenter. The difference between *E*_x_ and *E*_y_ increased with the indenter radius. The modulus in any direction increased with the indenter size. The reason for this was that more atoms contacted the tip of a bigger indenter [79]. Hence, the peak force was higher and further led to a higher modulus (Equations (4) and (5)). For example, *E*_x_ equals 17.85 GPa at *R* = 1.5 nm, or 27.02 GPa at *R* = 2.3 nm. *E*_y_ varies from 18.11 GPa at *R* = 1.5 nm to 31.21 GPa at *R* = 2.3 nm, or *E*_z_ from 27.51 GPa at *R* = 1.5 nm to 40.29 GPa at *R* = 2.3 nm.

## 4. Conclusions

In this study, the mechanical properties of calcium-silicate-hydrate (C-S-H) gel at atomistic level were evaluated using a nanoindentation approach via the MD simulations. The results were obtained with consideration of the effects of the essential factors, e.g., the sizes of sample and indenter, the maximum indentation depth, the loading speed, and the saturation of water in the gel samples. Some conclusions are drawn herein for a deeper understanding and application of the unique properties of the C-S-H gel on the nanoscale.

The elastic moduli in the three orthogonal directions indicate that the C-S-H sample behaves transverse isotropic with the material principal direction perpendicular to the C-S-H layers. The difference between the tensile and compressive moduli in the same direction demonstrates that the nanomaterial showed bi-modulus. It should be taken into consideration for precise upscaling numerical study. Moreover, the modulus of the C-S-H sample was sensitive to the loading speed; hence, the material had a high viscosity due to the relative motion of the water molecules between the silicon chains and the propagation of the stress wave in deformation. Specially, the radius of the indenter should not be lower than 2.0 nm. Otherwise, the slim indenter entered into the gap between two neighboring C-S-H layers and further led to a failure of test. Additionally, the moduli in a direction decreased with the increasing saturation of water in the sample. Polymerization occurred easier when saturation was lower. If the water saturation was too high, the silicates had little chance to be polymerized, which reduces the local stiffness.

The above conclusions help to develop the innovative cementitious materials from the bottom up. A model on a larger scale can be constructed with amorphous distribution of C-S-H gel particles, thus reducing the scale variability between simulation and experiment.

## Figures and Tables

**Table 1:** Nonbonding interaction parameters for different species in ClayFF Force Field [60].

Species *i*	Species *j*	$\varepsilon_{ij}$ (kcal/mol)	$\sigma_{ij}$ (nm)	Species *i*	Specie *j*	$\varepsilon_{ij}$ (kcal/mol)	$\sigma_{ij}$ (nm)
cah	cah	5.0298 × 10^−6^	0.5562	st	ob	5.3480 × 10^−4^	0.3234
cah	cao	5.0298 × 10^−6^	0.5564	st	obos	5.3480 × 10^−4^	0.3234
cah	st	3.0426 × 10^−6^	0.4432	st	o*	5.3480 × 10^−4^	0.3234
cah	ob	8.8410 × 10^−4^	0.4364	st	h*	0	0.1651
cah	obos	8.8410 × 10^−4^	0.4364	ob	ob	0.1554	0.3166
cah	o*	8.8410 × 10^−4^	0.4364	ob	obos	0.1554	0.3166
cah	h*	0	0.2781	ob	o*	0.1554	0.3166
cao	cao	5.0298 × 10^−6^	0.5567	ob	h*	0	0.1583
cao	st	3.0426 × 10^−6^	0.4434	obos	obos	0.1554	0.3166
cao	ob	8.8410 × 10^−4^	0.4366	obos	o*	0.1554	0.3166
cao	obos	8.8410 × 10^−4^	0.4366	obos	h*	0	0.1583
cao	o*	8.8410 × 10^−4^	0.4366	o*	o*	0.1554	0.3166
cao	h*	0	0.2783	o*	h*	0	0.1583
st	st	1.8405 × 10^−6^	0.3302	h*	h*	0	0

Note: cah: the interlayer calcium atoms, cao: the layered calcium atoms, st: silicon atoms, ob: bridging oxygen, obos: non-bridging oxygen, o*: water oxygen, h*: water hydrogen.

**Table 2:** Poisson’s ratio of C-S-H gel.

Parameters\Direction	x	y	z	Average
uniaxial tension	0.28	0.26	0.24	0.26
uniaxial compression	0.34	0.31	0.14	0.26
average	0.31	0.285	0.19	0.26
Reuss–Voigt–Hill approx.	-	-	-	0.27
Exp. data C-S-H gel	-	-	-	0.25

**Table 3:** Coordinates and the relative displacements of the observation points before and after loading.

	Before (nm)	After (nm)	Displacement (nm)		Before (nm)	After (nm)	Displacement (nm)
P1	X = 8.412	X = 8.045	−0.376	P3	X = 8.325	X = 7.761	−0.564
	Y = 4.372	Y = 4.361	−0.011		Y = 5.611	Y = 5.998	+0.387
	Z = 5.712	Z = 6.357	+0.645		Z = 4.415	Z = 4.639	+0.224
P5	X = 4.594	X = 4.726	+0.132	P7	X = 3.416	X = 2.766	−0.650
	Y = 8.266	Y = 8.184	−0.082		Y = 8.233	Y = 7.821	−0.412
	Z = 5.869	Z = 6.696	+0.827		Z = 4.331	Z = 4.085	−0.246
P9	X = 4.691	X = 4.738	+0.147	P11	X = 6.308	X = 6.817	+0.509
	Y = 6.003	Y = 6.257	+0.254		Y = 4.451	Y = 4.518	+0.067
	Z = 8.084	Z = 7.601	−0.483		Z = 7.999	Z = 7.533	−0.468
P2	X = 8.403	X = 7.589	−0.814	P4	X = 8.382	X = 7.541	−0.841
	Y = 4.423	Y = 4.472	+0.049		Y = 3.364	Y = 2.997	−0.367
	Z = 3.496	Z = 3.015	−0.481		Z = 4.423	Z = 4.102	−0.321
P6	X = 4.645	X = 4.596	−0.049	P8	X = 5.640	X = 6.080	+0.440
	Y = 8.285	Y = 7.883	−0.402		Y = 8.236	Y = 7.562	−0.674
	Z = 3.623	Z = 2.872	+0.751		Z = 4.323	Z = 3.869	−0.454
P10	X = 4.698	X = 4.747	+0.049	P12	X = 3.022	X = 2.545	−0.477
	Y = 3.342	Y = 2.991	−0.351		Y = 4.457	Y = 4.432	−0.035
	Z = 8.066	Z = 7.187	−0.879		Z = 8.082	Z = 7.743	−0.339

## Data Availability

The data presented in this study are openly available in https://data.mendeley.com/datasets/m3t8wk8pt5/1 (accessed on 31 July 2023).

## Supplementary Materials

The following supporting information can be downloaded at: https://www.mdpi.com/article/10.3390/nano13182578/s1. Movie S1-r=2nm-v=50ms-hmax=2nm-T=300k-s=100% during [0–80] ps.; Movie S2-r=2nm-v=50ms-hmax=2nm-T=300k-s=100% during [0–80] ps.
